# Bar, stripe and spot development in sand-dwelling cichlids from Lake Malawi

**DOI:** 10.1186/s13227-019-0132-7

**Published:** 2019-08-12

**Authors:** Laura A. Hendrick, Grace A. Carter, Erin H. Hilbrands, Brian P. Heubel, Thomas F. Schilling, Pierre Le Pabic

**Affiliations:** 1 Department of Biology and Marine Biology, University of North Carolina Wilmington, 5216 Randall Drive, Wilmington, NC 28403 USA; 2 Department of Developmental and Cell Biology, University of California, Irvine, 4109 Natural Sciences II, Irvine, CA 92697-2300 USA

**Keywords:** Cichlid, Pigment pattern, Melanophore, Xanthophore, Iridophore, Evolution, Pigmentation development, Neural crest, Morphogenesis

## Abstract

**Background:**

Melanic patterns such as horizontal stripes, vertical bars and spots are common among teleost fishes and often serve roles in camouflage or mimicry. Extensive research in the zebrafish model has shown that the development of horizontal stripes depends on complex cellular interactions between melanophores, xanthophores and iridophores. Little is known about the development of horizontal stripes in other teleosts, and even less is known about bar or spot development. Here, we compare chromatophore composition and development of stripes, bars and spots in two cichlid species of sand-dwellers from Lake Malawi—*Copadichromis azureus* and *Dimidiochromis compressiceps*.

**Results:**

(1) In *D. compressiceps,* stripes are made of dense melanophores underlaid by xanthophores and overlaid by iridophores. Melanophores and xanthophores are either loose or absent in interstripes, and iridophores are dense. In *C. azureus,* spots and bars are composed of a chromatophore arrangement similar to that of stripes but are separated by interbars where density of melanophores and xanthophores is only slightly lower than in stripes and iridophore density appears slightly greater. (2) Stripe, bar and spot chromatophores appear in the skin at metamorphosis. Stripe melanophores directly differentiate along horizontal myosepta into the adult pattern. In contrast, bar number and position are dynamic throughout development. As body length increases, new bars appear between old ones or by splitting of old ones through new melanophore appearance, not migration. Xanthophore and iridophore distributions follow melanophore patterns. (3) Metamorphic pigmentation arises in cichlids in a fashion similar to that described in zebrafish: melanophore progenitors derived from the medial route of neural crest migration migrate from the vicinity of the neural tube to the skin during metamorphosis.

**Conclusion:**

The three pigment cell types forming stripes, bars and spots arise in the skin at metamorphosis. Stripes develop by differentiation of melanophores along horizontal myosepta, while bars do not develop along patent anatomical boundaries and increase in number in relation with body size. We propose that metamorphic melanophore differentiation and migratory arrest upon arrival to the skin lead to stripe formation, while bar formation must be supported by extensive migration of undifferentiated melanophores in the skin.

## Background

The evolutionary diversification of body coloration is a fascinating subject because body color is at the center of multiple selective pressures, such as light protection, female mate choice and predation. As such, coloration likely plays an important role in the creation and maintenance of biodiversity, yet the genetic and developmental changes required for the evolution of new patterns with adaptive value remain largely unknown.

Various mechanisms underlie the diversity of colors and color patterns observed among vertebrates. The melanocyte is the only pigment cell type, or chromatophore, found in birds and mammals [1]. Melanocytes may produce different kinds of melanin pigment to produce colors ranging from yellowish (pheomelanin) to black (eumelanin). Other colors seen in birds and mammals result from food-derived pigments interacting with skin extracellular matrix [2, 3]. A greater diversity of chromatophore types underlies body coloration in reptiles, amphibians and fish, with seven types described in fish: xanthophores, erythrophores, iridophores, leucophores, melanophores, cyanophores and erythro-iridophores [4–7].

Vertebrate chromatophores are derived from an embryonic population of pluripotent and highly migratory cells called neural crest (NC) cells. These cells emerge from the dorsal neural tube toward the end of neurulation and migrate throughout the embryo to give rise to the skeletal and connective tissues of the face, neurons and glia of the peripheral nervous system and chromatophores, among many other derivatives [8]. Trunk NC cells migrate through two distinct routes previously thought to be associated with distinct fates. Chromatophore progenitors migrate along a “lateral” route between surface ectoderm and somite, while neuronal and glial progenitors migrate along a “medial” route between somite and neural tube [9–11]. Recent evidence has shown that chromatophores also arise from NC cells that travel along the medial route in zebrafish and give rise to melanophores and iridophores during metamorphosis or regeneration [12, 13]. It is unknown if this mechanism is conserved in other teleosts or other vertebrate clades.

The highly diverse patterns of chordate body colors result from the particular distribution of pigments and/or chromatophores and in many instances of their particular combinations and overlapping patterns in the skin. The numerous studies focusing on zebrafish horizontal stripe formation have made the developmental and genetic basis of this complex pattern the best understood of all animals. Three chromatophore types, black melanophores, yellow xanthophores and silver/blue iridophores make up the zebrafish stripe pattern. Dark stripes are composed of all three chromatophore types with melanophores providing darkness, while iridophores provide iridescence and blue hue or green hue when combined with xanthophores. Light interstripes are made of xanthophores overlaid on iridophores [14, 15]. This adult pattern arises gradually during metamorphosis, spanning the period of 3 to 6/7 weeks post-fertilization [16, 17]. A key finding in zebrafish stripe development is that the distribution of each chromatophore depends on interactions with other chromatophore types. Melanophores prevent the spreading of dense iridophores into dark stripe territory, iridophores and xanthophores promote melanophore numbers, yet xanthophores also prevent spreading of iridophores into dark stripe regions, maintain the integrity of melanophore stripes and prevent spreading of melanophores in light stripe regions [18–21]. It is thus crucial to consider all chromatophore types when attempting to understand body color development in other organisms. While horizontal stripes are commonly found in other teleost species, the development of other color pattern elements, such as vertical bars and spots, remains poorly studied. It is unknown if the cell–cell interactions described in danios exist in other genera and if so, how they generate complex body coloration. In addition, only a handful of studies have identified the types of genetic and developmental changes driving the evolution of such complex patterns [22–24].

The great diversity of color patterns seen in the cichlid species flocks of East African rift lakes makes them great models to elucidate the developmental basis of previously unexplored patterns. The young ages of the species assemblies found in lakes Victoria (500,000 years) and Malawi (4 million years; [25]) make them amenable to genetic mapping by allowing the production of fertile hybrids in laboratory conditions. Moreover, the presence of highly similar yet independently evolved color patterns in species from these lakes offers great opportunities to elucidate the mechanisms underlying convergent evolution [26] as recently demonstrated by Kratochwill et al. [23].

Here, we describe and compare the development of the distinct color patterns of two cichlid species from Lake Malawi, *Copadichromis azureus* and *Dimidiochromis compressiceps*, in order to identify the developmental differences underlying their divergent color patterns. The color pattern of *C. azureus* includes three dark spots and a series of vertical dark bars. The *D. compressiceps* pattern includes three horizontal dark stripes. Similar to zebrafish, we find that three pigment cell types underlie the color patterns of both species, melanophores, xanthophores and iridophores, with different distributions in *D. compressiceps* and *C. azureus* adults. The time of first appearance of each chromatophore type is similar in *D. compressiceps* and *C. azureus*. Development of the adult pattern begins during larval development, essentially defining the onset of metamorphosis. Xanthophores appear first in both species, followed by melanophores, and finally iridophores. *D. compressiceps* stripes lay directly on top of horizontal myotome boundaries, while *C. azureus* vertical stripes do not clearly correlate with patent anatomical boundaries. We demonstrate by in situ hybridization that migration of NC cells takes place between 42 h post-fertilization (hpf) and 54hpf in both species along the lateral and medial routes and that, similar to zebrafish, pigment progenitors migrate from the vicinity of the neural tube to the skin during metamorphosis.

## Results

### 1. Adult pigmentation in *C. azureus* and *D. compressiceps*

**Fig. 1:**
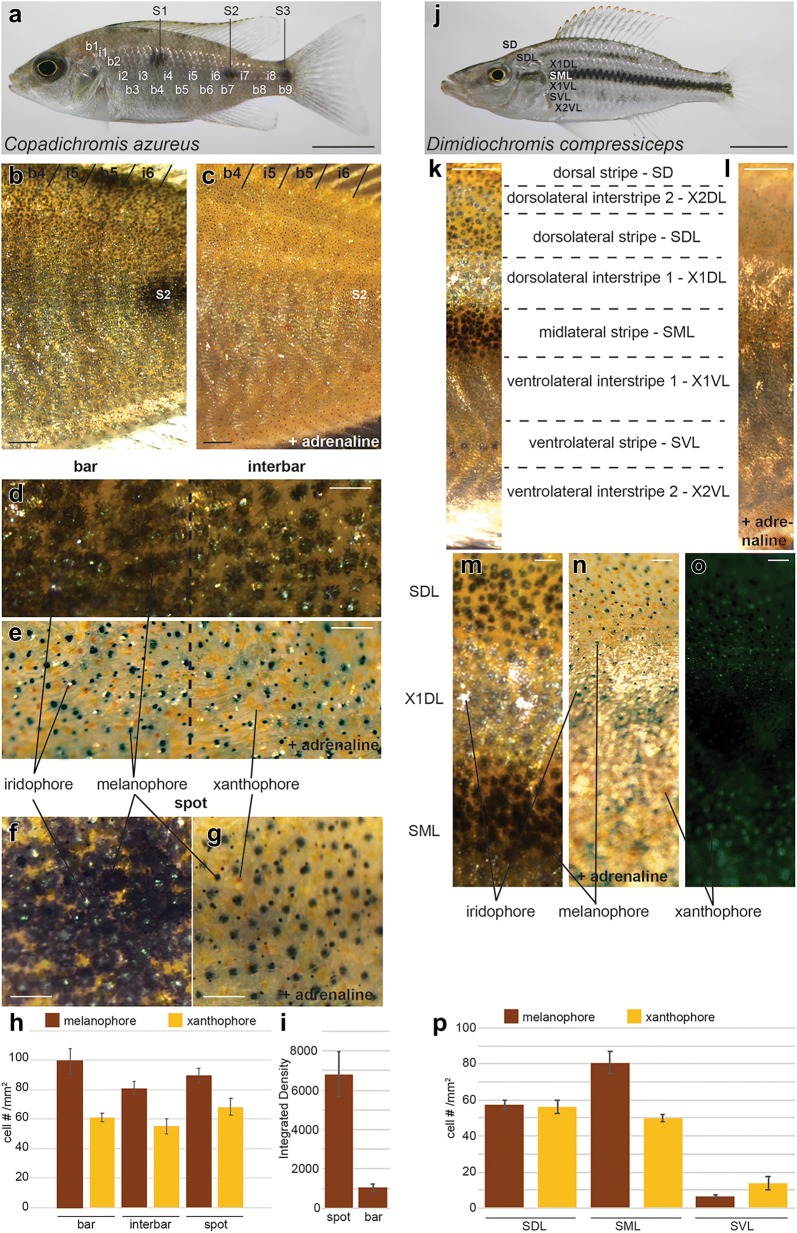
Adult pigmentation in *C. azureus* and *D. compressiceps*. **a** Vertical bars b1–9, interbars i1–8 and spots S1–3 in *C. azureus*. **b**, **c** Higher magnification images of bars/interbars and spots in *C. azureus* before (**b**) and after (**c**) adrenaline treatment. **d**, **e** Melanophores (black), xanthophores (orange) and iridophores (silver) in bars and interbars of *C. azureus* before (**d**) and after (**e**) adrenaline treatment. **f**, **g** Melanophores, xanthophores and iridophores of spot in *C. azureus* before (**f**) and after (**g**) adrenaline treatment. **h** Melanophore and xanthophore average densities in bars, interbars and spots (*n* = 3) in *C. azureus*. Error bars represent standard deviations. **i** Melanophore darkness in spot and bar expressed as integrated density. **j** Stripes and interstripes in *D. compressiceps* adult. **k**, **l** Higher magnification images of stripes and interstripes in *D. compressiceps* flank before (**k**) and after (**l**) adrenaline treatment. **m**–**o** Melanophores, xanthophores and iridophores of stripes SDL and SML and interstripe X1DL before (**k**, **m**) and after (**l**, **n**) adrenaline treatment. **o** Lack of xanthophores in interstripe X1DL visualized under fluorescent light (488 nm). **p** Melanophore and xanthophore average densities in SDL, SML and SVL (*n* = 3) in *D. compressiceps*. Error bars represent standard deviations. **a**, **j** Scale = 1 cm. **b**, **d**, **k**, **l** Scale = 1 mm. **d**–**g** and **m**–**o** Scale = 250 µm

The *C. azureus* body pigment pattern consists of the superimposition of two patterns commonly found in Lake Malawi, but also lakes Victoria and Tanganyika [26–28]: (1) an alternating series of dark bars and light interbars, and (2) three black spots (S1–3; Fig. 1a). Young adults have nine bars (b1–9) and eight interbars (i1–8; Fig. 1a), while more bars are observed on larger individuals. A series of five melanic patches variably aligned with vertical bars are present at the base of the dorsal fin. Individuals can modify the contrast between bars and interbars by changing bar darkness. A silver sheen covers the entire body with the exception of the spots, suggesting the widespread presence of iridophores. This pattern is seen in both males and females, although the silver sheen becomes metallic blue when males become dominant. In order to identify (1) what types of chromatophores are present in *C. azureus* and (2) how their particular arrangement creates the *C. azureus* pattern, we observed the skin of live individuals at various optical magnifications. Because pigment cells overlap one another in the skin, live specimens were photographed before and after l-adrenaline treatment to induce melanin granule aggregation in order to obtain a clear relationship between the abundance of particular chromatophores and the larger patterns observed on adult specimens: bars, interbars and spots. Three pigment cell types were identified: melanophores (dark brown/black), xanthophores (yellow-orange) and iridophores (silvery-white; Fig. 1b–g). All three chromatophore types are found in both the dark bars and the light interbars (Fig. 1d, e), as well as the spots (Fig. 1f, g). Melanophores were 23% more abundant in bars (99.4 cell/mm^2^) than interbars (81 cell/mm^2^; Fig. 1h), and cell size was larger in bars (Fig. 1d). Xanthophore abundance was 11% greater in bars (61.3 cell/mm^2^) than interbars (55.3 cell/mm^2^; Fig. 1d–e, h). Iridophores were generally overlaid on melanophores in spots, bars and interbars (Fig. 1d, f), where they seemed to provide greater cover although they could not be counted. Melanophore and xanthophore abundance was similar in spots as in bars (89.7 cell/mm^2^ and 68.3 cell/mm^2^; Fig. 1g, h), although melanophores were darker in spots (Fig. 1i).

The *D. compressiceps* body pigment pattern is also found on numerous cichlids from lakes Malawi, Tanganyika and Victoria and includes three horizontal dark stripes separated by light silvery interstripes (Fig. 1j, k; [27, 28]). An additional dark stripe runs along the dorsal midline (Fig. 1j, k). Dark stripes tend to disappear in dominant males as a metallic blue sheen develops over the entire body. As in *C. azureus*, melanophores, xanthophores and iridophores were identified by microscopy on live specimens before and after l-adrenaline treatment (Fig. 1k–o). All dark stripes contain the three chromatophores, while dorsal interstripes contain iridophores and melanophores, and ventral interstripes contain iridophores alone (Fig. 1k–o). Differences in melanophore density differentiate the stripes: melanophore density is the highest in the midline stripe (SML), intermediate in the dorsolateral (SDL) and dorsal (SD) stripes and lowest in the ventrolateral stripe (SVL), resulting in a discontinuous appearance (Fig. 1j–p).

### 2. Pre-metamorphic pigmentation development in *D. compressiceps* and *C. azureus*

**Fig. 2:**
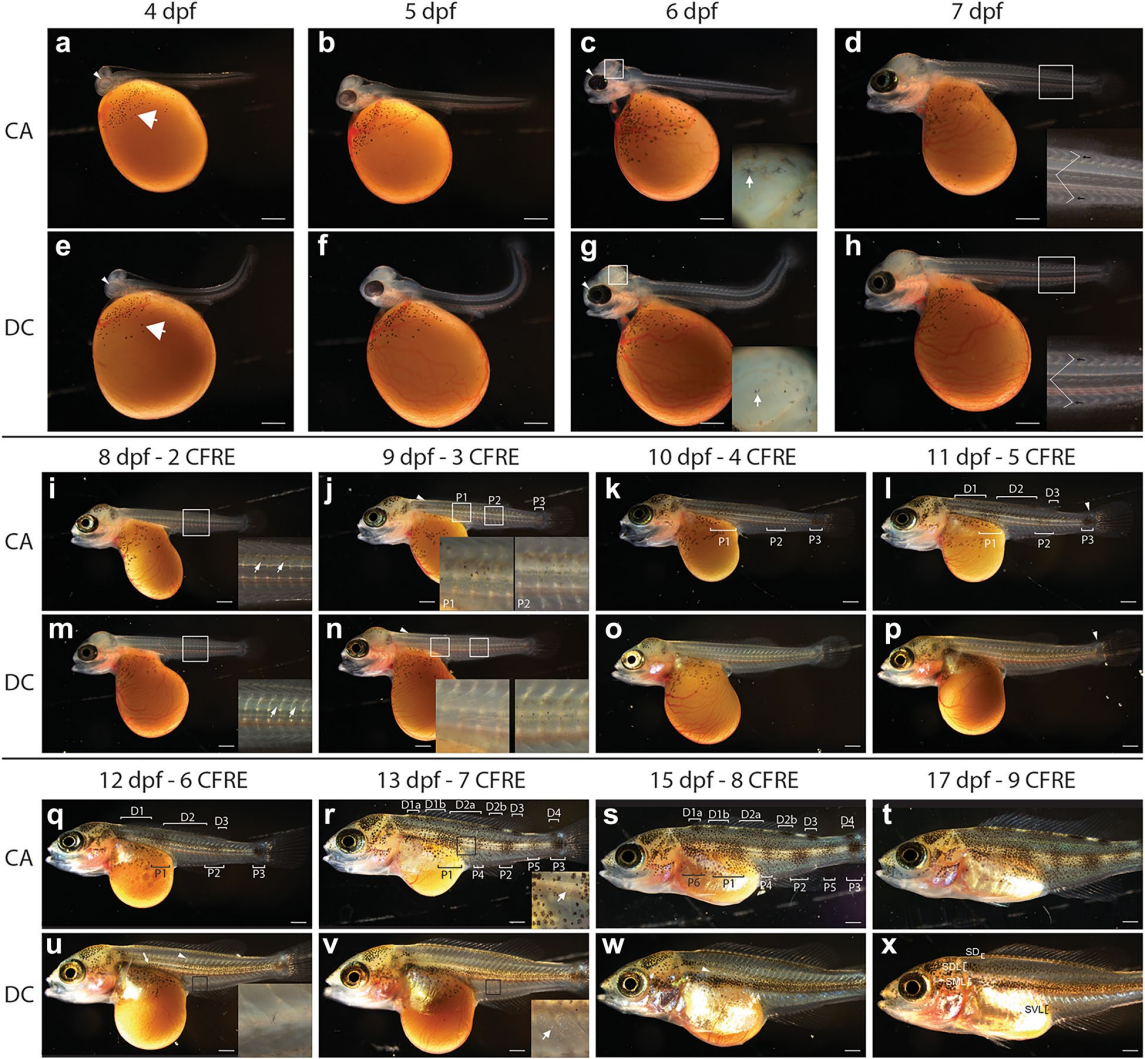
Larval development of pigmentation in *C. azureus* and *D. compressiceps*. **a**–**c**, **e**–**g** Pre-metamorphic development in *C. azureus* and *D. compressiceps* larvae. **a**, **e** Melanophores on the yolk (arrows) and melanization of the pigmented epithelium of the eye (arrowheads) in *C. azureus* and *D. compressiceps* at 4dpf. **c**, **g** Melanophores over the dorsal head in *C. azureus* and *D. compressiceps* (insets) and iridophores in the eye in *C. azureus* and *D. compressiceps* (arrowheads) at 6dpf. **d**, **h**–**x** Development of metamorphic pigmentation in *C. azureus* and *D. compressiceps*. **d**, **h** Myotome morphology at the onset of overt metamorphosis in *C. azureus* and *D. compressiceps* at 7dpf. **i**, **m** Melanophores over the dorsal neural tube in *C. azureus* and *D. compressiceps* (insets). **j**, **n** Skin melanophores at the base of the dorsal fin (arrowheads) and in *C. azureus* trunk and tail and *D. compressiceps* tail (insets). **k**, **l**, **o**, **p** Additional skin melanophores in *C. azureus* an *D. compressiceps*. **l**, **p** Iridophores in *C. azureus* and *D. compressiceps* in posterior caudal fin peduncle (arrowheads). **l** Dorsal melanophore patches D1–3 in *C. azureus*. **q** Dorsal patch D4 on caudal peduncle. **r** Dorsal patch D1 into D1a and D1b, dorsal patch D2 into D2a, D2b; lateral patches P4 and P5; and iridophores between melanophore patches (inset) in *C. azureus*. **s** Lateral melanophore patch P6 in *C. azureus*. **u** Melanophores at anterior portion of presumptive midlateral stripe SML (arrow), at presumptive dorsolateral stripe SDL (arrowhead) and presumptive ventrolateral stripe SVL (inset) in *D. compressiceps*. **v** Iridophores in presumptive ventrolateral interstripe 1 X1VL (inset). **w** Iridophores in presumptive dorsolateral interstripe 1 X1DL (arrowhead) in *D. compressiceps*. **x** Complete dark stripe pattern at late larval stage in *D. compressiceps*. Scale = 1 mm

In order to understand how each species-specific pigment pattern develops, live specimens from both species were imaged daily throughout embryonic and larval development, which ends after roughly 2 weeks of development when the yolk becomes fully depleted. Rates of development are extremely similar in *D. compressiceps* and *C. azureus*, and very similar to that described for the Nile tilapia [52]. Melanophores are the first pigment cells to differentiate in both species starting at 42hpf on the yolk (data not shown), followed by a 4-day period with minimal change to this pigmentation pattern (Fig. 2a–i, m), except in the eye where epithelial melanization and iridophores were first observed at 4dpf (Fig. 2a, e) and 6dpf (Fig. 2c, g), respectively.

**Fig. 3:**
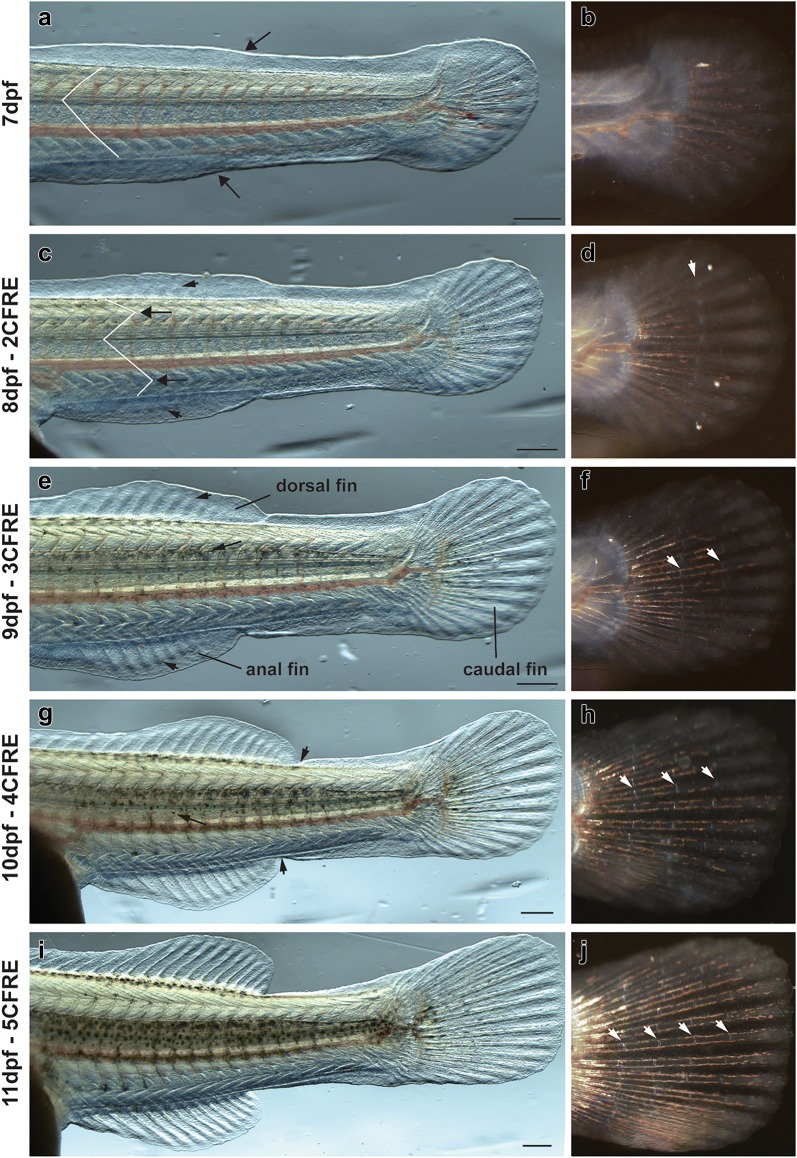
Morphological changes in median fins and myotomes during metamorphosis in *D. compressiceps*. **a** Bulges (arrows) in embryonic median fin prefigure the posterior ends of the dorsal and anal fin, respectively. “V” shape of myotomes indicated in white. **b** Unsegmented caudal fin ray elements. **c** Dorsal- and anal-fin ray condensations indicated by short arrows. Chevron shape of myotomes indicated by long arrows. **d** 2 caudal fin ray elements separated by one joint (white arrow). **e** Fin ray elements lengthen in dorsal fin and anal fin (short arrows). Melanophores appear on dorsal neural tube (long arrow). **f** 3 caudal fin ray elements separated by two joints (white arrows). **g** Dorsal and anal fins separated from caudal fin (short arrows). Melanophores appear in flank skin. **h** 4 caudal fin ray elements separated by three joints (white arrows). **i** Progressive disappearance of larval fin tissue from dorsal and ventral caudal fin peduncle. **j** 5 caudal fin ray elements separated by four joints (white arrows). The timing of metamorphosis is the same in *C. azureus* as in *D. compressiceps*. Scale = 500 µm

**Fig. 4:**
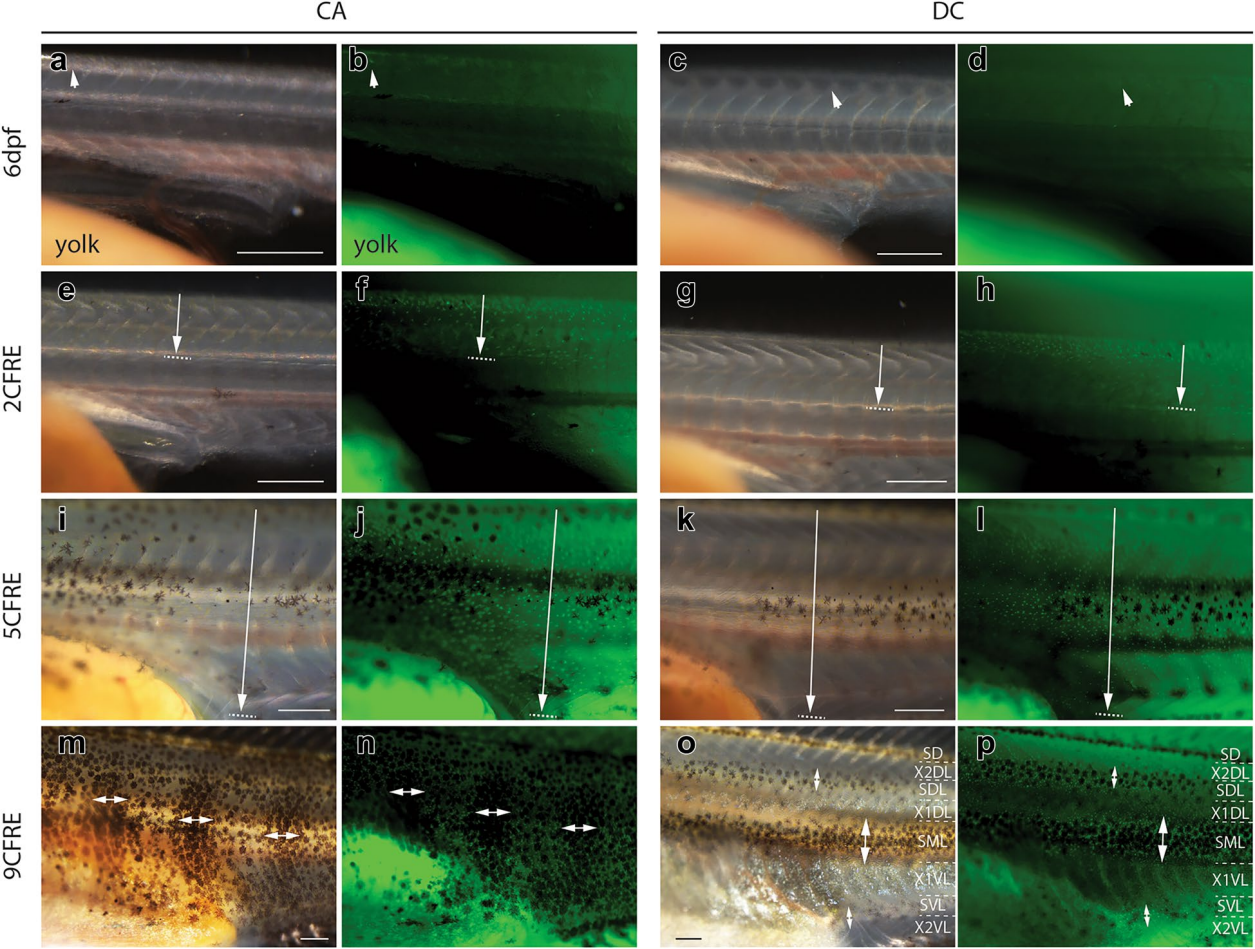
Xanthophore distributions in *C. azureus* and *D. compressiceps* larvae. **a**–**d** Xanthophores are restricted to the dorsal midline in *C. azureus* and *D. compressiceps* at 6dpf (arrows) as visualized with white light (**a**, **c**) or 488 nm fluorescent light (**b**, **d**). **e**–**h** Xanthophores extend from dorsal midline to horizontal myoseptum (arrow) in *C. azureus* and *D. compressiceps* as visualized with white light (**e**, **g**) or 488 nm fluorescent light (**f**, **h**). **i**–**l** Xanthophores extend from dorsal to ventral midlines (arrow) in *C. azureus* and *D. compressiceps* as visualized with white light (**i**, **l**) or 488 nm fluorescent light (**j**, **l**). **m**–**n** Xanthophore density is greater under melanophore patches (double-headed arrows) in *C. azureus* at 17dpf/9CFRE. **o**–**p** Xanthophores are largely restricted to dark stripes (double-headed arrows) in *D. compressiceps* at 17dpf/9CFRE. Scale = 500 µm

A new wave of skin pigmentation started at 6dpf (Fig. 2c, g), a day before overt morphological changes indicating the start of metamorphosis in both species such as separation of dorsal, caudal and anal fins and appearance of caudal fin ray elements (CFRE) (Fig. 3). Melanophores and xanthophores become visible on the dorsal head at 6dpf (Fig. 2c, g), followed by a progressive expansion of xanthophores alone along the dorsal trunk and tail at 7dpf (Fig. 4a–h). Xanthophore expansion proceeded ventrally over the next 3 days, reaching the lateral midline by 8dpf/2CFRE (Fig. 4e–h) and the ventral midline by 10dpf/5CFRE in both species (Fig. 4i–l). Following this full body coverage, xanthophore distribution became concentrated under bar melanophores in *C. azureus* (Fig. 4m, n) or stripe melanophores in *D. compressiceps* (Fig. 4o, p) by the end of the larval stage (9CFRE).

**Fig. 5:**
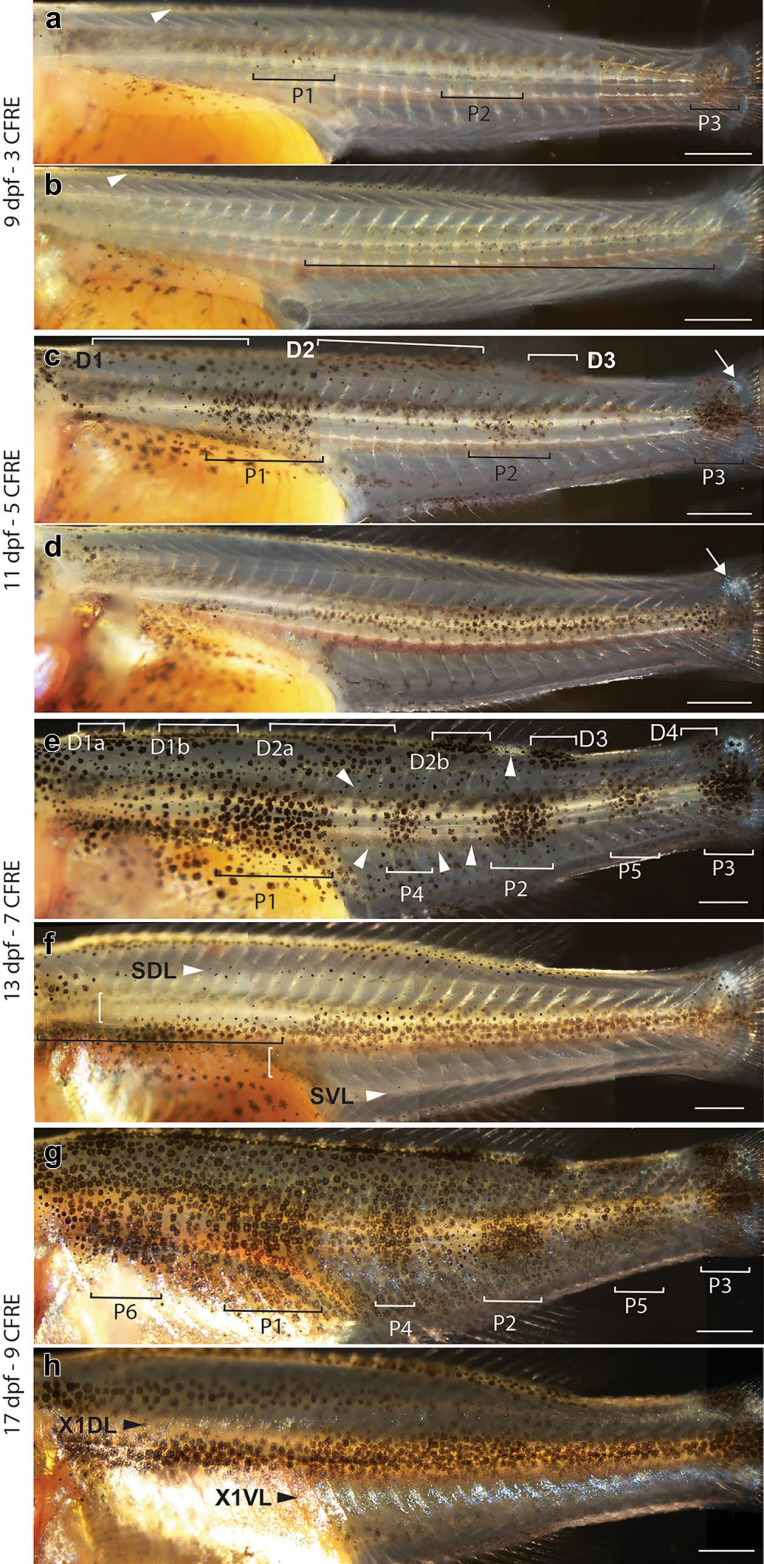
Melanophore and iridophore patterns in *C. azureus* and *D. compressiceps* larvae. **a** Melanophores in the flank in *C. azureus*—patches P1–3 and base of dorsal fin (arrow). **b** Melanophores along tail lateral midline and base of dorsal fin (arrow) in *D. compressiceps*. **c** Melanophore additions to and around P1–3 in *C. azureus*; Resolution of dorsal midline melanophore band into D1, D2 and D3. Appearance of iridophore patch on dorsal caudal fin peduncle (arrow). **d** Melanophore additions to tail-portion of dark stripe S0 in *D. compressiceps*. Iridophore patch on dorsal caudal fin peduncle (arrow). **e** Resolution of dorsal melanophore patches D1 into D1a and D1b, and of D2 into D2a, D2b; appearance of D4 on dorsal caudal fin peduncle. Appearance of lateral patches P4 and P5. Iridophore patches between dorsal and lateral patches (arrowheads). **f** Melanophores along trunk portion of SML in *D. compressiceps* (black bracket). Melanophores at presumptive SDL and SVL (arrowheads). Iridophores dorsal and ventral to horizontal myoseptum (white brackets). **g** New melanophores around populated areas. Appearance of lateral melanophore patch P6 in *C. azureus*. **h** Iridophores become abundant at interstripes X1DL and X1VL in *D. compressiceps*. Scale = 1 mm

Unlike xanthophores, metamorphic melanophores in the trunk (anterior to anus) and/or tail (posterior to anus) first appear deep in larvae of both species at 8dpf/2CFRE—on the dorsal side of the neural tube (Fig. 2i, m). These deep melanophores appear along the entire length of the neural tube in *C. azureus*, while they are restricted to the tail region in *D. compressiceps* at this stage. Skin melanophores are first observed the next day (9dpf/3CFRE) in both species—at the base of the dorsal fin, and at different regions along the lateral midline (Figs. 2j, n and 5a, b).

### 3. Metamorphic pigment pattern development in *C. azureus*

Three distinct patches (P) that prefigure the adult spots, but also the midparts of presumptive bars b4, b7 and b9 are observed in the skin at 9dpf/3CFRE along the lateral midline of *C. azureus*: P1 on the trunk, P2 on the tail, and P3 on the caudal fin peduncle (Figs. 2j and 5a). New melanophore appearance within and around these three patches leads to increased melanophore density as well as progressive expansion of each patch until 12dpf/6CFRE (Figs. 2j–l, q and 5a, c). By 13dpf/7CFRE new melanophore appearance leads to the formation of 2 new patches: P4 between P1 and P2, and P5 between P2 and P3 (Figs. 2r and 5e). Interestingly, appearance of these new melanophore patches takes place between differentiating iridophore streaks (Figs. 2r, s and 5e). At 15dpf/8CFRE, a new melanophore patch, P6, appears anterior to P1 (Fig. 2s). The progressive definition of *C. azureus* bars and interbars occurs concomitantly with the appearance of a series of dark and light patches along the dorsal midline. Similar to lateral midline patches, the first pigment cells appearing at the dorsal midline are melanophores concentrated in three melanophore patches (D1–3) at the base of the dorsal fin at 11dpf/5CFRE (Figs. 2l and 5c). New melanophores increase the cell density around these three centers, and iridophores start appearing in the two regions separating the patches by 6CFRE/12dpf (Fig. 2q). By 13dpf/7CFRE, D1 becomes resegmented into D1a and D1b, and D2 into D2a and D2b by new iridophore patches (Figs. 2r and 5e). One more melanophore dorsal patch (D4) flanked by iridophores also appears on the caudal fin peduncle (Figs. 2r and 5e). Some, but not all of these dorsal patches are dorso-ventrally aligned with the presumptive bars developing during the same period over *C. azureus* flanks.

**Fig. 6:**
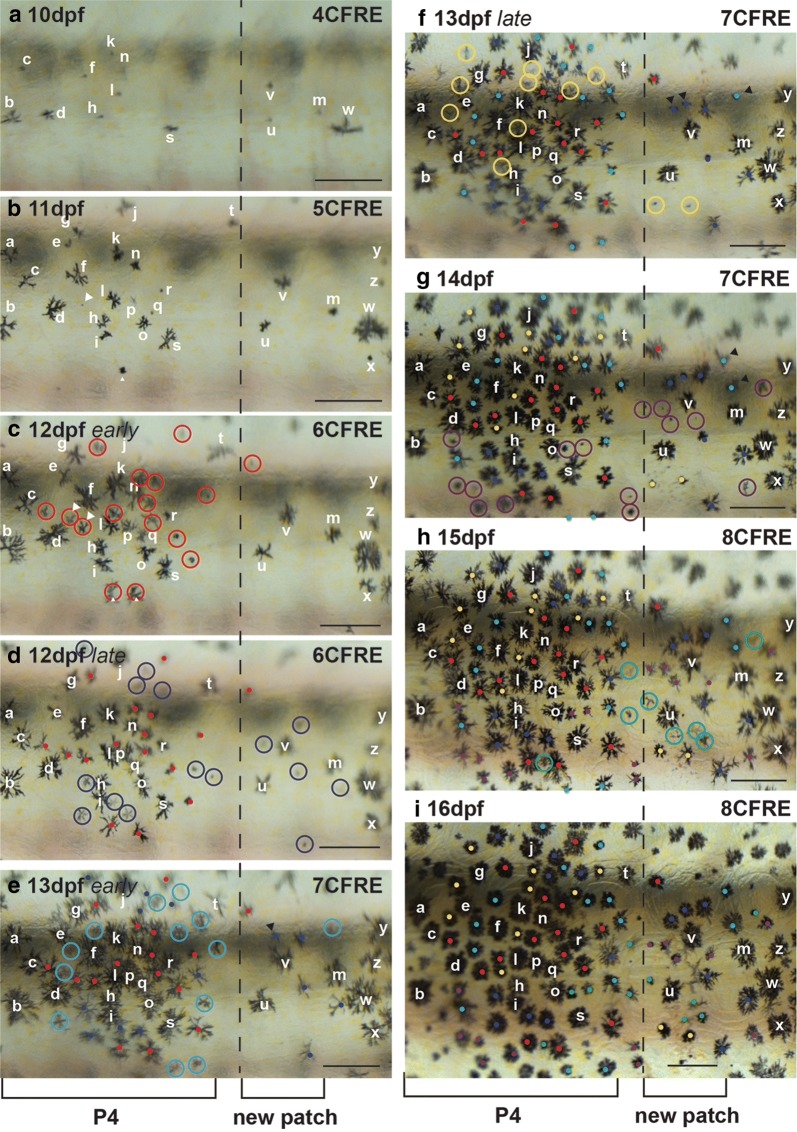
Timelapse recording of bar melanophore development in *C. azureus*. **a**–**i** Melanophores of presumptive lateral patch P4 (left) and a posteriorly appearing patch over 6 days of development. Melanophores present at 10dpf (**a**) and 11dpf (**b**) are identified by the same letters at all timepoints. **c** New melanophores are circled in red at this stage (12dpf early), and by red dots at subsequent stages. White arrowheads indicate daughter cells produced by melanophore mitosis. **d** New melanophores are circled in dark blue at this stage (12dpf late), and by dark blue dots at subsequent stages. **e** New melanophores are circled in light blue at this stage (13dpf early), and by light blue dots at subsequent stages. **f** New melanophores are circled in yellow at this stage (13dpf late), and by yellow dots at subsequent stages. Black arrowheads indicate daughter cells produced by melanophore mitosis. **g** New melanophores are circled in purple at this stage (14dpf), and by purple dots at subsequent stages. Black arrowheads indicate daughter cells produced by melanophore mitosis. **h** New melanophores are circled in green at this stage (15dpf), and by green dots at subsequent stages. **i** The relative positions of all melanophores examined remained largely unchanged from 10 to 16dpf. Scale = 250 µm

In order to determine whether lateral patch melanophores migrate ventrally from dorsal patches as previously proposed in Mesoamerican cichlids [29] and more generally what cell behaviors underlie stripe morphogenesis, 4 *C. azureus* larvae were imaged daily from 10dpf/4CFRE to 18dpf/9CFRE at high magnification to follow individual melanophore behavior during bar formation (Fig. 6). Most melanophores did not move after appearing in the skin (Fig. 6a–i), and few mitoses were observed. Bars are thus shaped by the addition of new melanophores in *C. azureus*.

### 4. Metamorphic pigment pattern development in *D. compressiceps*

In contrast to *C. azureus*, the posterior half of the midlateral stripe in *D. compressiceps* first appears at 9dpf/3CFRE as a continuous stripe of melanophores along the lateral midline in the tail region (posterior to anus; Figs. 2n, 5b). Melanophores are also observed at the base of the dorsal fin at this stage, prefiguring the dorsal stripe (Fig. 5b). Melanophore density increases each day by the addition of new melanophores to posterior SML and SD (Figs. 2o, p, u–x and 5b, d, f, h). Melanophores forming the anterior half of SML appear dorsal to the yolk at 12dpf/6CFRE (Fig. 2u), along with those forming the dorsal (SD) and ventrolateral stripes (SVL). New melanophore additions through later stages increase cell density in each stripe without changing the pattern prefiguring the adult stripes (Fig. 2u–x).

Interstripe iridophores appear dorsal and ventral to the midlateral stripe at 13dpf/7CFRE (Figs. 2v and 5f), and new iridophore additions through later stages give rise to interstripes X1DL and X1VL (Figs. 2x and 5h). The adult *D. compressiceps* pigment pattern is essentially complete by the end of the larval period (17dpf/9CFRE) (Figs. 2x and 5h).

### 5. Timing of chromatophore progenitor development

Based on the observed onset of adult pigmentation at metamorphosis in *D. compressiceps* and *C. azureus*, we next asked what are the embryonic origins of metamorphic chromatophores in these cichlids. In zebrafish, metamorphic melanophore and iridophore progenitors arise from stem cell pools associated with dorsal root ganglia, which are derived from ventral/medial route NC cells and replace embryonic/larval pigment cells [12, 13]. These progenitors migrate to the skin along sensory axons traveling between myotomes [13]. In contrast, larval xanthophores are derived from dorsal/lateral route NC cells and remain in the skin at metamorphosis and into adulthood [17].

In order to determine when chromatophore progenitors arise and migrate in *D. compressiceps* and *C. azureus*, we conducted in situ hybridization to detect the expression of NC- and chromatophore-specific markers at various developmental stages in *D. compressiceps* and *C. azureus* embryos. *FoxD3* and *sox10* label premigratory and migratory NC cells, respectively [30–33]. *Kit*-*l*, *mitfa*, *tyr* and *tyr*-*p* label melanophore progenitors [34–36]; *csf1ra*, *gch* and *xdh* label xanthophore progenitors [18, 37, 38]; *pnp4 and bmp4* label iridophore progenitors [39]. In addition, *ednrb1a* labels all three chromatophore types in zebrafish [40], as well as migratory NC cells in mammals [41].

**Fig. 7:**
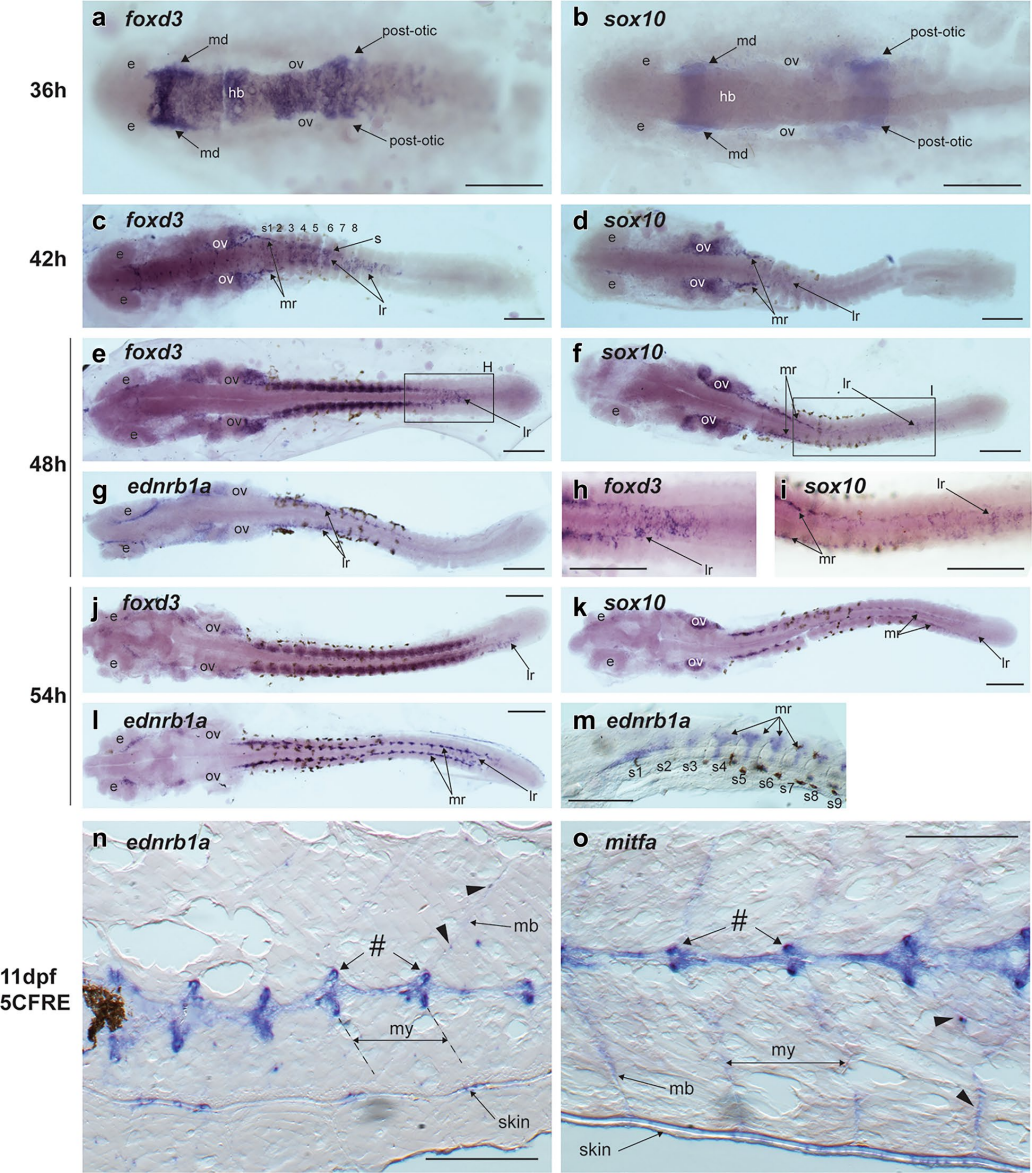
Expression of neural crest (NC) cell- and pigment progenitor markers during embryonic development and later in metamorphosis in *C. azureus*. **a**–**m** Whole mount in situ hybridizations (ISH), anterior to the left, dorsal view (**a**–**l**) and lateral view (**m**). **a**, **b** *foxd3* and *sox10* expression in presumptive hindbrain, mandibular- and post-otic NC streams at 36hpf. **c**–**f**, **h**, **i** *foxd3* and *sox10* expression in trunk NC cells migrating through the lateral- and medial routes at 42 and 48hpf. *Foxd3* is also expressed in somites. **g** *ednrb1a* expression in trunk NC cells migrating through the medial route at 48hpf. **h**–**i** Higher magnifications of *foxd3* and *sox10* expression in trunk NC cells migrating through the lateral- and medial routes at 48hpf. **j**–**k** *foxd3* and *sox10* expression in trunk NC cells migrating through the lateral- and medial routes at 54hpf. *Foxd3* expression is also detected in somites. **l** *ednrb1a* expression in trunk NC cells migrating through the medial route at 54hpf. **m** Higher magnification of *ednrb1a* expression in trunk NC cells migrating through the lateral route at 48hpf (somites 1–9 numbered). **n**–**o** ISH on cryosections of 11dpf/5CFRE in *C. azureus* larvae. *Ednrb1a* and *mitfa* expression is detected at high levels in segmented structures located dorsal to the neural tube (#), and at lower levels along myotome boundaries (arrowheads) and in the skin. *e* eye, *hb* hindbrain, *lr* lateral NC route, *mb* myotome boundary, *md* mandibular NC stream, *mr* medial NC route, *my* myotome, *ov* otic vesicle, *post-otic* post-otic NC stream, *s* somite. Scale = 250 µm

Gene expression patterns were first examined at 36, 42, 48, 54 and 60hpf based on previously described timing of cranial NC migration in another cichlid, the Nile Tilapia [42]. *Sox10* and *foxD3* expression was detected at 36 and 42hpf in *C. azureus* (Fig. 7a–d) and *D. compressiceps* (data not shown). At 36hpf, *foxD3* is expressed in the hindbrain and in premigratory cranial NC cells, as well as two streams of NC cells just beginning to migrate bilaterally, which we interpret as the mandibular NC stream (directly posterior to the eye) and the post-otic NC stream (posterior to otic vesicle) (Fig. 7a). *Sox10* expression was also detected in the hindbrain and cranial NC streams, albeit at lower intensity (Fig. 7b).

At 42hpf, *foxd3* expression was still detected in all three cranial NC streams, as well as in two distinct cell populations in the anterior trunk. One population was found ventrally between the neural tube and the first two somites, which we interpret as NC cells migrating along the medial/ventral route. A second population consisted of individual cells on top of the neural tube, which we interpret as NC cells migrating along the lateral route (Fig. 7c). At this and later stages, laterally migrating NC cells were present further posteriorly than medial/ventrally migrating NC cells, consistent with an earlier onset of lateral migration during the anterior–posterior sequence of trunk NC migration, as described in other vertebrates, including zebrafish [43]. *foxD3* was also detected in the medial parts of somites 3–7, as previously described in zebrafish [44]. *Sox10* expression at these stages was restricted to the pre- and post-otic streams of cranial NC but was also detected in medially migrating NC at the levels of somites 1–2, and in laterally migrating NC cells further posteriorly, similar to *foxd3* (Fig. 7d).

Trunk expression of *foxD3* and *sox10* at subsequent stages (48, 54hpf) was detected in successively more posterior streams of NC cells. A notable difference between the medial and laterally migrating populations was the duration of *foxd3* and *sox10* expression: expression of both was maintained in NC streams migrating medially but downregulated in streams migrating laterally (Fig. 7e, f, h–k). *Ednrb1a* expression was also detected at 48 and 54hpf in both species. In the trunk, *ednrb1a* expression labeled medial NC streams similar to *foxd3* and *sox10* (Fig. 7g, l, m), and also lateral NC streams at 54hpf (Fig. 7l). Expression of *foxd3*, *sox10* or *ednrb1a* decreased by 60hpf and was no longer detected at 66 or 72hpf, and none of the other markers listed above produced detectable signals from 36 to 72hpf.

The undifferentiated progenitors of zebrafish metamorphic melanophores and iridophores reside at dorsal root ganglia until metamorphosis, when they migrate along nerve axons and between myotomes to the skin [13]. In order to determine whether similar pigment progenitors exist in cichlids, we conducted in situ hybridization on cryosections at premetamorphosis (96hpf) and metamorphosis stages (11dpf/5CFRE) using *ednrb1a* as a pan-chromatophore marker and *mitfa* as a melanophore marker. Expression of both genes was first detected at 11dpf/5CFRE (Fig. 7n, o) along the neural tube, between myotomes, and in the skin, consistent with a migration of chromatophore progenitors from the vicinity of the neural tube to the skin during metamorphosis. No difference in *mitfa* or *ednrb1a* expression was detected between *D. compressiceps* and *C. azureus* at these stages.

## Discussion

### Chromatophore distribution relative to anatomical landmarks

How do spatial cues instruct pigment cells to differentiate in particular regions of the embryo or larva? Previous findings in zebrafish have shown that metamorphic melanophore progenitors (melanoblasts) travel from dorsal root ganglia to the skin along axons between myotomes [13]. Here, we provide evidence for a similar mechanism in cichlids. This suggests a simple mechanism for horizontal stripe formation along horizontal myosepta: melanoblasts migrating to the skin along horizontal myosepta could give rise to horizontal stripes by differentiating in the skin upon arrival. In contrast, additional melanoblast migration would be required in the skin for vertical bar formation.

**Fig. 8:**
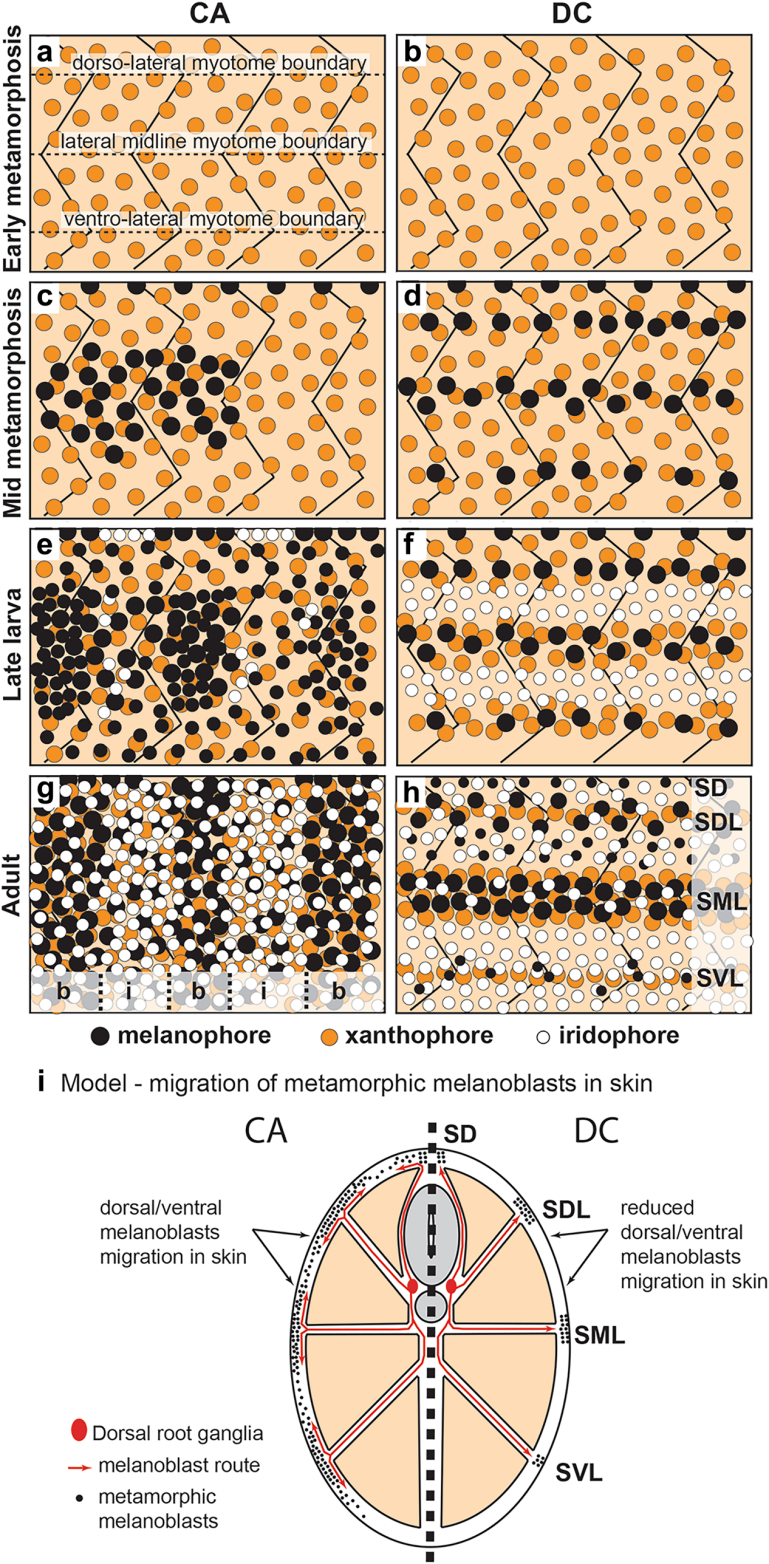
Summary of pigment pattern developmental differences in *C. azureus* and *D. compressiceps*. **a**, **b** Xanthophores cover the body in both *C. azureus* and *D. compressiceps* at the onset of metamorphosis. **c**, **d** Melanophores appear in the skin of *C. azureus* and *D. compressiceps* during metamorphosis, with a similar strip of metamorphic melanophores at the base of the dorsal fin in both *C. azureus* and *D. compressiceps*. **c** Patches of metamorphic melanophores appear on the flank skin in *C. azureus* and grow in size by new melanophore addition in and around each patch. **d** Metamorphic melanophores appear along the dorsolateral-, lateral midline- and ventrolateral myotome boundaries in *D. compressiceps*. **e**, **f** Chromatophore distribution at the end of the larval period in *C. azureus* and *D. compressiceps*. **e** Melanophore patches elongate dorso-ventrally and become resolved into bars flanked by iridophores in *C. azureus*. New bars appear by appearance of new melanophores. Xanthophore density increases under bar melanophores. **f** New melanophores appear largely along already formed horizontal stripes in *D. compressiceps*. Xanthophores increase in density under stripe melanophores and disappear from interstripe regions. Iridophores appear in interstripe regions. **g**, **h** Pigment cell patterns in *C. azureus* and *D. compressiceps* adults. **g** In *C. azureus* bars are composed of dense melanophores lying on dense xanthophores and under loose iridophores. Melanophore densities are lower in interbars than in bars, while iridophore density is greater. Xanthophore distribution is similar in bars and interbars. **h** In *D. compressiceps* stripes are composed of melanophores and xanthophores in varying densities (greatest in SML, intermediate in SDL, lowest in SVL). Ventral interstripes are composed of iridophores exclusively, while dorsal interstripes also contain loose melanophores. **i** Model of differential migration of metamorphic melanoblasts in the skin of *C. azureus* (left) versus *D. compressiceps* (right). We propose that migratory potential is a major developmental difference in metamorphic melanoblast behavior between *C. azureus* and *D. compressiceps*. Under this model, *C. azureus* metamorphic melanophores do migrate dorsally and ventrally once they reach the skin, while skin migration is minimal in *D. compressiceps*, which results in melanophore positioning over horizontal myotome boundaries and formation of horizontal stripes

*Dimidiochromis compressiceps* dark stripes are clearly overlaid on horizontal myosepta: SML melanophores differentiate along the lateral midline myoseptum, stripe SD along the dorsal midline myoseptum, and stripes SDL and SVL along the dorsal and ventral lateral myosepta, respectively (Fig. 8b, d, f, h). In contrast, *C. azureus* vertical bars or spots arise from a progressively greater number of lateral melanophore patches that progressively elongate dorso-ventrally from new melanophores (Fig. 8a, c, e, g), without clear relationships with underlying myosepta. A major developmental difference resulting in the different color patterns of *C. azureus* and *D. compressiceps* may thus be in the migratory behavior of melanoblasts in the skin (Fig. 8i). Accordingly, the *D. compressiceps* pattern may result from the differentiation of melanoblasts into melanophores with limited migratory potential once they reach the skin, while the *C. azureus* pattern would require the migration of melanoblasts in the skin before differentiation into melanophores (Fig. 8i), since differentiated melanophores are distributed throughout the skin regardless of myoseptum position.

### Developmental regulation of bar number in old-world and new-world cichlids

Vertical bars similar to those found in *C. azureus* are found in many cichlid species from both Africa and the Americas. Bar ontogeny was described in 40 species of neotropical cichlids of the subfamily Cichlasomatinae with various numbers of bars at adults stages [29]. Interestingly, the authors found that 9 vertical bars (including 2 in the head) develop first in all species examined, followed by species-specific bar divisions or fusions. Similar to *C. azureus*, bars form progressively starting with an initial pattern of lateral and dorsal midline melanophore clusters in Cichlasomatinae cichlid larvae. Dorsal and/or ventral migration of melanophores away from the clusters was reported to lead to the formation of bars. Our observation of individual melanophores in the skin of individual *C. azureus* larvae through several days of development did not show such migration of differentiated melanophores. Instead, supported exit points of metamorphic melanoblasts at horizontal myosepta suggest dorsal and ventral migration of melanophores through the skin in the undifferentiated state to result in the vertical bar pattern of *C. azureus*.

The timing of metamorphosis was also recently experimentally shown to modulate the number of vertical bars in a Mesoamerican cichlid, the convict cichlid *Amatitlania nigrofasciata* [45]. Consistent with a perturbation of metamorphosis [46], it was found that the timing and level of thyroid signaling activity affect (1) the timing of larval to juvenile coloration, and [2] the number of vertical bars. Individuals under elevated thyroid hormone levels from embryo to adult stages start pigmentation metamorphosis earlier than untreated individuals and develop 7 bars instead of 9, while individuals with lower thyroid hormone levels start pigmentation metamorphosis later and end up with 12 bars.

How does the timing of metamorphosis affect the number of bars? A common developmental pattern reported in barred Mesoamerican cichlids and here in *C. azureus* is the resolution of a few broad melanophore patches into numerous thin bars from larval to adult stages. In *C. azureus* new bars appear in growing larvae by new melanophore appearance in the skin—not rearrangement of already present melanophores. New bars form between old ones that have become spaced apart through body growth. Body growth also correlates with bar splitting—it creates new space between bar melanophores, which is filled by new melanophores within daughter bars but remains vacant between daughter bars. These observations suggest that increase in physical space along the anterior–posterior axis is a positive regulator of bar number. The increase in bar number associated with delayed metamorphosis in the Mesoamerican cichlid *A. nigrofasciata* [45] also suggests that physical space at the time of first bar appearance is also a critical regulator of bar number. *A. nigrofasciata* individuals with delayed metamorphosis developed roughly the same number of bars as *C. azureus* at juvenile stages; however, these bars were thinner and thus more widely spaced, perhaps leaving more room for later-appearing bars.

### Genetic determinants of cichlid color patterns

The ontogeny of zebrafish body coloration is by far the best understood of all vertebrates because of the numerous mutant screens that have led to the identification of genes required for melanophore, xanthophore and iridophore development, but also for the interactions among and between these cell types and with their environment [47, 48]. However, this understanding is largely restricted to the development of horizontal stripes. The diversity of cichlid color patterns in closely related species and their amenability to genetic mapping makes them a great model system to explore the developmental and genetic underpinnings of pigment pattern evolution in vertebrates. Research efforts have so far been far greater to identify the genetic factors regulating cichlid pigmentation than their developmental basis. Although most naturally occurring trait variants are under the control of multiple genes, as illustrated in Albertson et al. [49], two instances of monogenic traits were discovered in East African cichlids. Cis-regulatory variants affecting *pax7a* expression were found to be associated with the development of the orange-blotch camouflage pattern in Mbuna cichlids of Lake Malawi [50, 51]. More recently, a cis-regulatory variant affecting *agrp2* expression was shown to determine the presence/absence of the horizontal dark stripe found in lake Victoria cichlids *Haplochromis sauvagei* and *Pundamilia nyererei* [23]. These authors also showed that difference in *agrp2* expression reliably predicted the presence/absence of dark stripe in cichlids from other East African Lakes, thus demonstrating a clear case of convergence under the control of a single gene [23].

Here, we identified potential developmental differences underlying pigment pattern divergence in *D. compressiceps* and *C. azureus*. Identification of the loci regulating these developmental differences will constitute the next step toward a broader understanding of the developmental evolution of body coloration in vertebrates.

## Conclusion

The three same chromatophore types make up *D. compressiceps* and *C. azureus* body coloration: melanophores, iridophores and xanthophores. In *D. compressiceps,* stripes are made of dense melanophores underlaid by xanthophores and overlaid by iridophores. Melanophores and xanthophores are either loose or absent in interstripes, and iridophores are dense. In *C. azureus,* spots and bars are composed of a chromatophore arrangement similar to that of stripes but are separated by interbars where density of melanophores and xanthophores is only slightly lower than in stripes and iridophore density appears slightly greater.

Differences in pigmentation between *C. azureus* and *D. compressiceps* arise at metamorphosis when melanophores appear in the skin as arrangements prefiguring stripes, bars or spots. In *D. compressiceps,* stripe melanophores differentiate along horizontal myosepta and the adult pattern is essentially in place by the end of the larval period. In contrast, the number of bars developing in *C. azureus* larvae is smaller than in adults. Bars appear as a series of lateral melanophore patches that progressively elongate dorso-ventrally by new melanophore additions. New bars appear either between old ones, or through splitting of old ones. The place of new melanophore appearance, not migration, determines new bar formation. Increase in xanthophore density in developing bars and stripes follows that of melanophores. Iridophores appear in interstripes after stripe formation, while their appearance in interbars is concomitant with bar formation.

Analysis of neural crest migration by ISH indicates that melanophore progenitors migrate from the vicinity of the neural tube to the skin along myosepta at metamorphosis, as previously described in zebrafish [13]. Time-lapse analysis of bar development during metamorphosis shows that melanophores do not migrate after differentiation. We thus propose that metamorphic melanophore differentiation and migratory arrest upon arrival to the skin lead to stripe formation, while bar formation must be supported by extensive dorsal and/or ventral migration of undifferentiated melanophores in the skin.

## Methods

### Animal care

All animals were reared and euthanized following protocols approved by the IACUC at the University of North Carolina, Wilmington. *D. compressiceps* and *C. azureus* brood stocks were purchased from a pet store and maintained separately at 28 °C in 55- or 75-gallon tanks. Adults were fed Northfin Food Cichlid Formula 3 mm sinking pellets. Fertilized eggs were collected from mouth-brooding females a few hours after fertilization. Embryos were then staged under a microscope, reared in cichlid egg tumblers and imaged daily until the end of the larval period (yolk depletion). Embryos and larvae were staged following Fujimura and Okada, 2007 [52].

### Image capture

For imaging of live specimens, individuals were anesthetized in tank water using Tricaine (MP Biomedicals) following protocols approved by the IACUC at the University of North Carolina, Wilmington and immobilized on a drop of 3% methyl cellulose (Alfa Aesar). l-Adrenaline treatment (Alfa Aesar) was carried out under the same conditions at a concentration of 4.5 mg/mL. Chromatophore density averages reported in Fig. 1h, p were obtained from regions representative of each pattern in *C. azureus* (*n* = 3) or *D. compressiceps* (*n* = 3). To measure and compare melanophore darkness between bars and spots (Fig. 1i), images were [1] converted to greyscale and [2] inverted in Photoshop. Integrated density was then measured in Fiji, with darker regions displaying higher integrated density values than lighter regions. For time-lapse imaging of *C. azureus* larvae, 4 specimens were imaged daily from 10dpf/4CFRE to 18dpf/9CFRE at high magnification to follow individual melanophore behavior during bar formation. Each specimen was anesthetized daily as described above, imaged and returned to its tank. Daily image capture was determined to be adequate to follow new melanophore appearance after multiple-daily image captures showed barely noticeable changes to the melanophore pattern. Melanophore migratory activity was assessed by recording [1] the position of melanophores relative to stable landmarks (myosepta) and [2] the relative positions of closest neighbors over the entire duration of the time-lapse imaging. The replacement of a single melanophore by 2 melanophores in successive time points was interpreted as a mitosis. All observations and images were made using a dissecting microscope Leica M165FC equipped with Planapo 1.6X M-series objective and Prior L200 fluorescent light source. Image capture was made with a Leica DFC7000T camera controlled by the LAS X software. Chromatophore counts were performed in FIJI. Image processing was performed in Adobe Photoshop.

### In situ hybridization

Embryos and larvae were fixed and processed for whole mount in situ hybridization as described in Le Pabic et al. [53]: embryos were fixed in 4% PFA for 3 days at 4 °C, rinsed in PBT and transferred to 100% methanol at − 20 °C for storage. For cryosections, embryos and larvae were processed as described in Albertson et al. [54]. In situ hybridization probes were generated from RT-PCR amplified regions of *C. azureus foxd3*, *sox10*, *kit*-*l*, *mitfa*, *tyr*, *tyr*-*p*, *csf1ra*, *gch*, *xdh*, *pnp4*, *bmp4*, *ednrb1a* cDNAs using the primers listed in Table 1. mRNA was extracted from deyolked 54hpf *C. azureus* embryos. In situ hybridization was conducted using antisense probes prepared from pGem-t-easy vectors (Promega) containing cDNA fragments corresponding to *C. azureus foxd3*, *sox10*, *kit*-*l*, *mitfa*, *tyr*, *tyr*-*p*, *csf1ra*, *gch*, *xdh*, *pnp4*, *bmp4* and *ednrb1a*.

**Table 1:** Primers

Gene	Forward primer	Reverse primer	Amplicon length (bp)
*foxd3*	TAAAGCCGCCTTACTCCTAC	GCGAGGAAGAGGAGGCAC	654
*sox10*	CTGGGTCAGGAAGCGAGG	GCTCGAATGGGCGTAGTAT	683
*kit*-*l*	TGTGTCCATTTCCTGCTGTT	TTCCTCCGTGATCTGACCTT	647
*mitfa*	TCGCATTAAAGAGCTGGGAA	ACTGCAGTCATGTTTCACAG	819
*tyr*	TGGACGCAACTCCCTTAATC	GCAATCAGAGCTCCCAGAAT	708
*tyr*-*p*	GTTTCCTGACCTGGCACA	CGGTCACAAACATCTCCAAG	711
*csf1ra*	TCCCACCTTCAGCAAAATCT	TTCATCAGAGGCTGCTCTTC	479
*gch1*	CCAACCTACATGAACCAGCT	GCACACGTCTAATGTTGCTC	861
*xdh*	GGCAGCCTGTCATAACCATA	CACATCGGTCATCCAGTTCT	716
*pnp4*	GACGAATACCAGAAGACAGCT	CCTCGTAACTCTTCACCACC	730
*bmp4*	CACACTGCTGCATATGTTCG	CCGTAATTGGGAAGTCGTTC	531
*ednrb1a*	CCACAGAAATCCGAGACACT	GACAGATGCCATGTTGATGC	854

## Data Availability

The datasets used and/or analyzed during this study are available from the corresponding author on reasonable request.
